# Effects of different modalities of inspiratory muscle training as an add-on to conventional treatment of patients with chronic obstructive pulmonary disease (COPD): study protocol for a randomized controlled trial

**DOI:** 10.1186/s13063-019-3271-1

**Published:** 2019-04-24

**Authors:** Catharinne Angélica Carvalho de Farias, Lucien Peroni Gualdi, Selma Bruno da Silva, Verônica Franco Parreira, Dayane Montemezzo, Vanessa R. Resqueti, Guilherme A. F. Fregonezi

**Affiliations:** 1 Laboratório de Desempenho PneumoCardioVascular e Músculos Respiratórios, Departamento de Fisioterapia, Universidade Federal do Rio Grande Do Norte (UFRN), Natal, Rio Grande do Norte Brazil; 2 PneumoCardioVascular Lab/HUOL, Empresa Brasileira de Serviços Hospitalares - EBSERH), Universidade Federal do Rio Grande do Norte (UFRN), Natal, Rio Grande do Norte Brazil; 3 Faculdade de Ciências da Saúde do Trairi, Universidade Federal do Rio Grande do Norte (UFRN), Santa Cruz, Rio Grande do Norte Brazil; 4 Centro de Reabilitação Cardíaca e Metabólica, Empresa Brasileira de Serviços Hospitalares (EBSERH), Universidade Federal do Rio Grande do Norte (UFRN), Natal, Rio Grande do Norte Brazil; 5 Departamento de Fisioterapia, Universidade Federal de Minas Gerais (UFMG), Belo Horizonte, Minas Gerais Brazil; 6 Centro de Ciências da Saúde e do Esporte, Universidade do Estado de Santa Catarina (UDESC), Florianópolis, Santa Catarina Brazil

**Keywords:** COPD, Training, Respiratory muscle training, Pulmonary rehabilitation

## Abstract

**Background:**

Chronic obstructive pulmonary disease (COPD) leads to peripheral and respiratory muscle dysfunctions. Nowadays, inspiratory muscle training can be geared toward strength or endurance gains. This study aims to investigate the effects of an inspiratory muscle training (IMT) protocol using different therapeutic modalities to be implemented in pulmonary rehabilitation programs. The effects of IMT on exercise capacity were considered as the primary endpoint, and the effects of IMT on inspiratory muscle function, health-related quality of life, and daily physical activity level were considered as the secondary outcomes.

**Methods:**

This study is a blinded-investigator randomized controlled clinical trial. Sixty subjects will be randomly allocated into three groups: (1) pulmonary rehabilitation (PR) associated with inspiratory muscle training without any load (PRWIMT), (2) PR associated with inspiratory muscle training with a linear load (PRIMTLL), and (3) PR associated with inspiratory muscle training with isocapnic voluntary hyperpnea (PRIMTIVH). The protocol will be performed 5 days a week (3 days with supervision) for 10 weeks. The study will assess anthropometric data, lung function, respiratory muscle strength, and functional capacity by the Incremental Shuttle Walking Test and the Six-Minute Walk Test, lung volumes during the submaximal endurance test, peripheral muscle strength of the upper and lower limbs, dyspnea, and quality of life related to health, before and after the training protocol. Normality will be tested using the Kolmogorov-Smirnov test, and variables will be compared by two-way analysis of variance. The significance level was set at *p* < 0.05. Ethics approval was obtained from the Institutional Ethics Committee in Research (1.663.411). The study results will be disseminated through presentation at specific scientific conferences and publication in peer-reviewed journals.

**Discussion:**

The different IMT protocols used in our study will be able to guide respiratory therapists to understand and to include in conventional PR programs the most effective respiratory muscle training type in subjects with COPD.

**Trial registration:**

Brazilian Clinical Trials Registry, RBR-94v6kd. Registered on 11 March 2017.

**Electronic supplementary material:**

The online version of this article (10.1186/s13063-019-3271-1) contains supplementary material, which is available to authorized users.

## Strengths and limitations of the study

The study has the following strengths:

- The study is an investigator-blinded randomized controlled trial designed to aid in better understanding further effects that inspiratory muscle training (IMT) may add to conventional pulmonary rehabilitation (PR) programs.
- The subjects will be reevaluated after the protocol by an evaluator blinded to the intervention protocol allocation to describe the effects of different IMT modalities on exercise capacity.

One limitation of the study is that the training protocol will be partially performed at home by the studied subjects without clinical supervision.

## Background

Chronic obstructive pulmonary disease (COPD) is a chronic, preventable, and treatable disease that is characterized by persistent respiratory symptoms and airflow limitations; the primary triggering factor is significant long-term exposure to noxious particles or gases, which produce airway and/or alveolar airway changes [1]. The disease presents extrapulmonary manifestations such as skeletal muscle dysfunction, which is an important prognostic factor that justifies exercise intolerance in these patients [2].

In addition to pharmacological treatment, it is recommended that individuals with COPD take part in PR programs. Nowadays, PR is defined as an embracing intervention based on the complete evaluation of the patient followed by specific therapies that include but are not limited to training, education, and behavior changes, aiming to improve the physical performance and psychological status of individuals with chronic respiratory disease and to promote long-term adherence and behavior changes [3].

IMT may generate strength increase in patients with COPD; however, studies have shown that the training may overload these muscles when not well performed [4]. Although decades have passed with several studies about IMT in patients with COPD being conducted, there are still doubts regarding IMT efficiency and its influence on exercise tolerance in COPD subjects. Studies have shown that IMT does not determine exercise performance improvement in COPD, or that there is a weak correlation between respiratory muscle changes due to IMT and exercise performance [5].

The literature describes two modalities of respiratory muscle training: isocapnic voluntary hyperpnea, which consists of a training that requires the individual to perform voluntary hyperventilation during a certain period of time, maintaining an isocapnea [6]; and threshold valves of pressure loading, which require the individual to perform inspirations or expirations against resistance [7]. This device allows inspirations and expirations to be forced by the device, which causes selective strengthening of the respiratory muscles and mobilizes the entire rib cage. Studies show that therapy with isocapnic voluntary hyperpnea reduces dyspnea and improves exercise capacity and the quality of life of subjects [8]. Currently, the market has electronic IMT valves that seem to provide some benefits in relation to volume increase during training compared to mechanical valves; however, these effects have not yet been clinically demonstrated [9]. There have been, to date, disagreements over the effects of IMT in individuals with COPD [8].

Due to different results on the effect of IMT in COPD subjects, the aim of this study will be to investigate the effects of an IMT protocol to be included in PR programs. The first outcome will be the effects of the training on exercise capacity. Training on inspiratory muscle function (strength and resistance), state of health, and dyspnea level will be considered as secondary outcomes. We believe that the different protocols used in our study are able to guide physiotherapists with regard to the most effective type of IMT, as well as to elucidate the effects of IMT on exercise tolerance, to identify if there is any repercussion on strength and the resistance of the respiratory muscles when subjected to different training types. In addition, the best IMT modality can be included in conventional PR programs, allowing optimization of the therapeutic management of patients with COPD. One also hopes that these results will endorse adjustments to the modalities used to strengthen respiratory muscles, as we believe that a well-implemented therapeutic program can reduce morbidity/mortality rates in these individuals as well as improve their quality of life.

## Methods

### Study

This is a blinded randomized controlled clinical trial. The PR program will be performed by a respiratory therapist. Initial and final assessment will be performed by another previously trained respiratory therapist who will be blinded to the allocation groups in accordance with the Consolidated Standards of Reporting Trials (CONSORT) [10] and Standard Protocol Items: Recommendations for Interventional Trials (SPIRIT) [11].

### Subjects

Individuals with a clinical diagnosis of COPD according to the Global Initiative for Chronic Obstructive Lung Disease (GOLD) being treated at the Ambulatory Pulmonology department of University Hospital Onofre Lopes (HUOL)/Empresa Brasileira de Serviços Hospitalares (EBSERH) will be invited to participate in the study. All selected individuals will be assessed at the PneumoCardioVascular Lab HUOL/EBSERH to confirm disease stage. Patients in clinical follow-up with a specialized pulmonology physician and with use of adequate bronchodilation (modified Medical Research Council [mMRC] score ≥ 2/4), who are aged between 40 and 80 years, living in the city of Natal, RN/Brazil, not using oxygen therapy or presenting disease exacerbation in the last 3 months, and not practicing regular physical activity in the last 6 months will be eligible to participate. Subjects presenting musculoskeletal comorbidities that impair the subject’s gait, peripheral oxygen saturation (SpO_2_) < 90% during the Six-Minute Walk Test (6MWT), hypertensive subjects without control medication as well as those presenting with a hypertensive peak (> 140/90 mmHg) [12] for more than 3 consecutive days, those with an intellectual understanding impairment that interferes with the evaluation tests, or those who stop the therapeutic program, miss activity for more than 1 week, or miss reevaluation will be excluded from the study.

### Study design

**Fig. 1:**
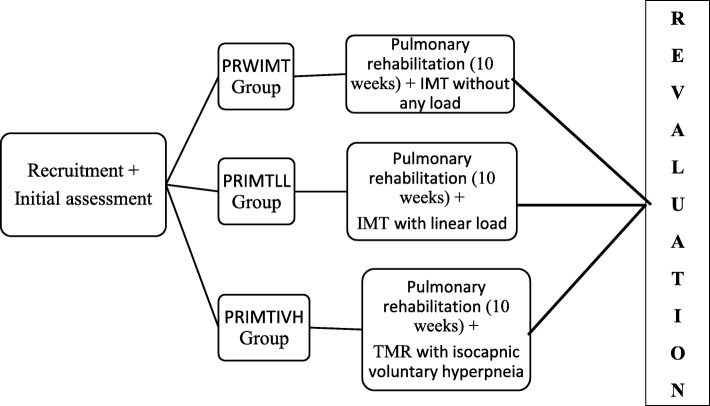
Flow diagram of intervention protocols

**Fig. 2:**
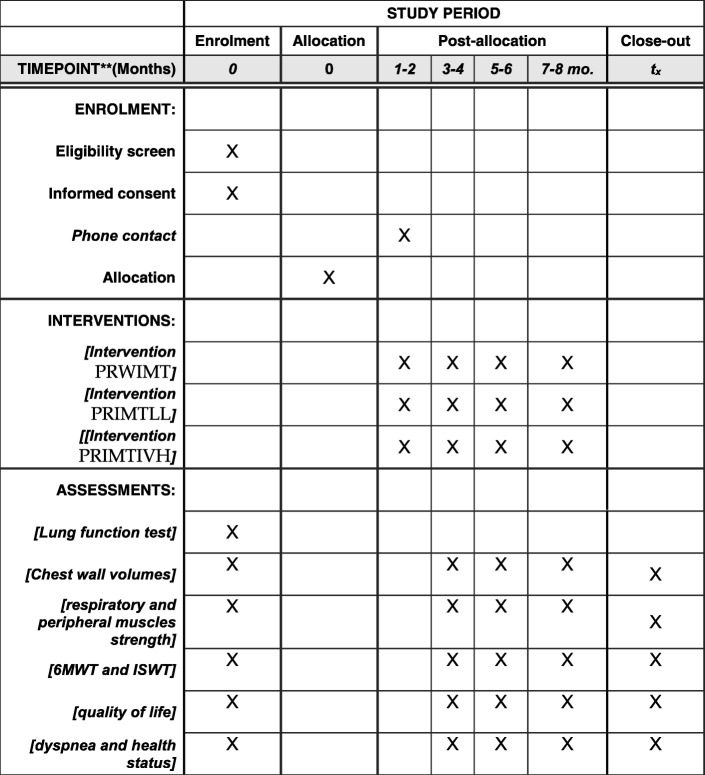
Schedule of the study

After recruitment, subjects will be invited to attend the PneumoCardioVascular and Respiratory Muscle Performance Laboratory HUOL/EBSERH to perform an initial evaluation. The assessment will be performed by a previously trained evaluator blinded to the intervention allocation group and will include anthropometry, lung function, respiratory muscle strength, exercise capacity, lung volumes associated with the submaximal endurance test, peripheral muscle strength (upper and lower limbs), dyspnea, and impact of COPD on a person’s life. After evaluation, a secure web-based randomization system (randomization.com) will be used to allocate subjects into three groups: the PRWIMT group will perform PR and IMT without any load; the PRIMTLL group will perform PR and IMT with a linear load, and the PRIMTIVH group will perform PR and IMT with isocapnic voluntary hyperpnea. The web-based randomization firstly generates a list of numbers from 1 to 60. These numbers are randomized into the study groups to which the subjects will be numerically allocated in order of arrival. After the intervention period, a blind evaluator to the intervention groups, as represented in Fig. 1, the flow diagram of the intervention protocols, will reevaluate all cases, according to the schedule of the study in Fig. 2. The SPIRIT checklist is provided as Additional file 1.

### Intervention

#### Pulmonary rehabilitation program and respiratory muscle training

The PR protocol will be performed three times a week for 10 consecutive weeks. All activities will be supervised by a previously trained respiratory therapist in a room with a controlled temperature set between 23 and 26 °C. All individuals will be instructed to perform the rehabilitation program 5 days a week — 3 days with supervision and 2 without supervision — for at least one hour a day, according to the recommendations of the guideline on PR in adults [13]. Subjects will receive orientation to fill in the unsupervised exercise diary regarding type of exercise, duration, and frequency as well as for symptoms referenced during the activity using the modified Borg scale (from 4 to 6) to limit the intensity of the exercise [14].

All three groups will submit to a conventional rehabilitation program consisting of aerobic exercise on a treadmill. For the first week, the subjects will perform 20 min of activity. A resting period lasting 2 min maximum will be allowed whenever requested by the subject. The training time will be increased after the 5th week to reach 30 min of daily activity [15]. Aerobic exercise will be performed on a treadmill, with a velocity corresponding to 70% of the maximum velocity achieved in the Incremental Shuttle Walking Test (ISWT) [16]. Ten percent of the starting time (2–3 min) of the training will be at a speed of 40% of the maximum speed reached in the ISWT for heating, 80% of the training time (17–25 min) will be at the target speed (70% ISWT), and the remaining 10% of the time will be cooling, with the same speed as for heating, reducing the speed to zero at the end of the training period [17, 18]. The physical activity training will be interrupted whenever the degree of dyspnea sensation reaches 4–6 (intense) on the Borg scale [14] or the heart rate (HR) exceeds 85% of the individual maximum HR value (220 – age). If the saturation drops (SpO_2_ < 90%) [19] during the exercise, we will administer supplemental oxygen at minimal flow with a nasal cannula to maintain higher than 90% saturation. The patients will be monitored every 5 min. Participants will be instructed to maintain aerobic activities on days that they are not performing the supervised PR program. Strength training for lower and upper limb muscles will be performed through a program consisting of 3 sets of 12 repetitions, initially using 60% of 1 maximal repetition (1RM), and this load will be increased to 70% by the 5th week and 80% in the 8th week of training [20]. For the upper limbs the training will be directed to the biceps, triceps, pectoral, and deltoid muscles, and for the lower limbs, the training will be for quadriceps and triceps femoris [3]. Guidance in energy conservation techniques as well as educational speeches regarding self-management of the disease will be provided [13]. The PRWIMT group will perform PR and IMT without any load; the PRIMTLL group will perform PR and IMT with a linear load; the PRIMTIVH group will perform PR and IMT with isocapnic voluntary hyperpnea.

##### PRWIMT protocol

The PRWIMT protocol consists of conventional PR as previously described. Individuals will also perform a placebo IMT using a POWERbreathe® device (POWERbreathe International Ltd., Southam, UK) without any load for 3 cycles of 12 repetitions.

##### PRIMTLL protocol

The PRIMTLL protocol consists of conventional PR and the use of POWERbreathe®. POWERbreathe KH1 (POWERbreathe International Ltd.) will be used for IMT. This equipment is based on resistance training to inspiration and consists of a mouthpiece coupled to the equipment, which will electronically generate inspiratory resistance to the exercise. POWERbreathe KH1 performs an automatic estimate of the patient’s load necessity at the beginning of each session [21]. Respiratory muscle training will be performed using 35% of maximal inspiratory pressure (MIP), increasing 5% every week until reaching 80% of MIP at the 10th week, which will be maintained until the end of the protocol. The MIP will be accessed weekly to adjust the training load percentage. Subjects will perform 3 cycles of 12 repetitions as proposed by Huang et al. [22].

##### PRIMTIVH protocol

The PRIMTIVH protocol consists of conventional PR and IMT using an STMedical® device (SpiroTiger, Chamonix Mont Blanc, France). Respiratory muscle training in voluntary isocapnic hyperventilation mode will be performed with the STMedical® device Pulmonary Rehabilitation Maximal Voluntary Hyperpnea (PRMVH), consisting of a portable unit with a pouch and a base station. A two-way piston valve is connected to a rebreathing bag, which maintains a constant isocapnic fraction of carbon dioxide [23]. Individuals will receive training with duration up to 20 min, one minute of training and one minute of rest, where they will be encouraged with instructions such as “breathe faster.” The training respiratory rate chosen will be calculated as 35-fold forced expiratory volume in the first second (FEV_1_), so that ventilation corresponds to 50–60% of maximal voluntary ventilation (MVV) [24]. They will start with a respiratory rate (RR) between 26 and 30 bpm lasting for 1 min, until completion of 20 min of training with the target RR. The volume of the rebreathing bag will be based on the forced vital capacity (FVC) of the patient, starting with a rebreathing bag size approximately equivalent to 50–60% of the FVC [25, 26].

### Outcome measures

#### Anthropometric assessment

Patients will be assessed for their height and body weight through an anthropometric scale coupled with a Filizola stadiometer (Filizola PL200®, São Paulo, Brazil) and a maximum capacity of 200 kg in order to calculate body mass index (BMI). As proposed by Quetelet (1835), BMI has been used by the World Health Organization (WHO) since 1997 as a reference measure of obesity worldwide and is expressed as the weight in kilograms divided by the square of the height in meters (weight/height^2^) [27].

#### Lung function test

Lung function will be assessed using a KoKo DigiDoser spirometer (Nspire Health, Longmont, CO, USA). Technical procedures, acceptability, and reproducibility will follow the recommendations of the American Thoracic Society/European Respiratory Society (ATS/ERS) [28]. Subjects will perform between three to eight maneuvers, and the best three will be recorded with variability less than 5% or 200 ml. The test will be performed before and after the use of an inhalation bronchodilator. Absolute and predicted values of FEV_1_, FVC, and FEV_1_/FVC will be considered and analyzed using a previously published equation for the study population [29].

The dynamic muscular endurance test (MVV) will also be performed. The dynamic test will be performed by MVV during 15 s (MVV_15s_) using a KoKo DigiDoser spirometer (Nspire Health). The individual will be positioned in a seated position on a bench with a nose clip and will be instructed before the beginning of the test to “breathe as fast and deep as possible.” During the test, active stimulation will be carried out with the cadence “pull and drop, pull and drop” to maintain a constant and regular rhythm with the same volume and frequency during the whole test. The maneuver will be assessed by three tests of 15 s. The highest value will be considered the best test. A difference less than 10% among the tests will be set [28, 29].

#### Maximal respiratory pressure, sniff nasal inspiratory pressure (SNIP), and sustained maximum inspiratory pressure (SPImax)

Respiratory muscle strength will be measured using a MicroRPM digital manometer (NEPEB-LabCare/UFMG, produced in Belo Horizonte, Brazil). Technical procedures, acceptability, and reproducibility will follow the recommendations of the ATS/ERS (2002) [30]. To obtain MIP, the individual will be instructed to perform one maximal expiration (close to residual volume, RV) followed by a maximal inspiration (close to total lung capacity, TLC). To obtain maximal expiratory capacity (MEP), the individual will be instructed to perform a maximal inspiration (close to TLC) followed by a maximal expiration (close to RV) [31]. A maximum of five maneuvers will be performed for each evaluation, and the highest value will be considered so that the difference among the maneuvers does not exceed 10% among the best three tests. Obtained values will be compared to reference values for the Brazilian population [32].

The SNIP, or sniff, test will be performed with one nostril occluded by a nose clip connected to the manometer through a catheter measuring approximately 1 mm. The maneuver will be performed from functional residual capacity (FRC). The individual will be instructed to perform a slow sustained expiration followed by a maximal sniff (inspiration) through the contralateral nostril. All subjects will perform 10 maneuvers with a 30-s interval among them [33]. The highest maneuver will be compared to previously described equations to determine reference values for the studied population [34].

To measure SPImax, the same manometer will be used. The manometer will be connected to a diver through an acrylic piece with an air leak orifice of 2 mm and a 6-mm occlusion orifice. The manometer will communicate with software (Manovac 3.0-A), whereby the data will be accessed after being collected [35–37]. Individuals will be instructed to breathe quietly close to tidal volume through the nozzle connected to the manometer for three consecutive breaths. The evaluator will give the following verbal command: “Push the air out, push the air into ... ”. The next steps are as follows: (1) slow exhalation, close to the residual volume at the verbal command “Push all the air out ... ”; (2) signaling the end of the total and slow exhalation by the patient, shown by raising the left arm; (3) obstruction of the occlusion hole by the evaluator, and (4) achieving a maximum inspiratory effort, near to total lung capacity, which will be sustained as long as possible, at the command: “Take a deep breath! And hold it, hold it ... ... ... hold it” [30, 37, 38]. The test will be considered complete when the subject performs at least three acceptable efforts, which means with no air leakage between the lips and the mouthpiece and/or nose clip, and of these, two reproducible efforts (with variation equal to or less than 10% of the test, with the variable area under the pressure-time curve value, since it was not the last test). Up to eight inspiratory efforts will be performed, with a one-minute interval between the tests [39].

#### Assessment of chest wall volumes

Analysis of respiratory kinematics integrated to signals of lung volumes, flow, pressure, and respiratory muscle activity will be assessed during a spirogram (one minute of quiet breathing [QB] followed by a maximum respiratory cycle, 20 s of QB, a maximum voluntary ventilation for 15 s, and one minute of QB). All signals will be integrated using Optoelectronic Plethysmography (OEP) System; BTS, Milan, Italy). The evaluation of flow and pressure generated in the airways will be performed using a Series 0–800 lpm pneumotachograph (flow measurement head), interconnected to a Series RSS100HR amplifier (Research Pneumotach System, Hans Rudolph, Inc.; Shawnee, KS, USA). The chest wall volumes and its compartments (rib cage pulmonary, rib cage abdominal, and abdomen) will be assessed by OEP System. This system allows the computational kinematic analysis of the three dimensions of the chest wall complex through 89 optical sensors placed on the chest wall. The sensors, small hemispheres (5 mm in diameter) coated with a reflective substance, will be circumferentially placed on the three horizontal regions of the chest wall between the collarbone and the superior iliac spine. Sensors will be placed in five vertical columns on the anterior and posterior regions and in two additional columns on the axillary line [40–43]. The limit between the pulmonary and abdominal rib cage will be assumed at the xiphoid process level, and the limit between the abdominal rib cage and abdomen will be assumed as the lower anterior costal margin and lower posterior costal margin. Coordinates of the sensors will be assessed by using a kinematic configuration system in eight photosensitive cameras (four on the front region and four on the back region) with an acquisition frequency of 60 Hz. Based on the captured images, the volumes of the chest wall will be computed by a triangulation surface. Respiratory pattern volume, flow, and time will be considered for the analysis. Additionally, operational volumes (end inspiratory volume and end expiratory volume) will be evaluated [44].

#### Peripheral muscle strength

Upper limb muscle strength will be obtained by hand dynamometry on the dominant side using Jamar equipment (Sammons Preston, Bolingbrook, IL, USA). Three reproducible tests will be performed (≤ 5%), and the highest obtained value will be considered for analysis using a previously described reference equation [45]. Lower limb muscle strength will be assessed using the maximal repetition (MR) test of the dominant limb on an extension board (405 Embreex Physicus, Brazil) with a load capacity of 50 kg [46].

#### Six-Minute Walk Test (6MWT) and Incremental Shuttle Walking Test (ISWT)

The 6MWT will be performed following the technical procedures described by the ATS [47]. Respiratory rate, heart rate, and peripheral oxygen saturation (SpO_2_) by portable pulse oximeter (Nonim Medical, Inc., 2500A, Plymouth, MN, USA) and systemic arterial pressure (SAP) using an analogue tensiometer (Becton Dickinson - BD®, Brazil) and a stethoscope (Littmann® classic II, 3M, Saint Paul, MN, USA) will be assessed before, immediately after, and 2 min after the end of the test. The subjective symptoms of dyspnea and fatigue of lower limbs will be evaluated with the modified Borg scale, from 0 to 10 [14]. Heart rate and SpO_2_ will be monitored every minute during the test. At the end of the test, the total distance walked will be calculated, and the equation of Iwama et al. [48] will be used to calculate the predicted walking distance during the test. To evaluate the maximum work (Wmax) performed during the 6MWT, the equation proposed by Hill et al. [49], which is based on the subject’s body weight, will be used. The predicted values of distance will be calculated according to Iwama et al. [48].

The ISWT will be performed according to Singh et al. [50]. The test will be interrupted by subject exhaustion or if the individual is not able to keep the proposed speed. Respiratory and cardiac frequency, SpO_2_, SAP, and subjective symptoms of dyspnea and lower limb fatigue will be measured before and after the test with the modified Borg scale [14, 51]. The equation of Probst et al. [52] will be used to calculate predictive walking distance in the study.

#### Dyspnea and health status

Perceived dyspnea will be evaluated by the modified Medical Research Council (mMRC) scale, which has five different items in which the subject chooses the one that corresponds to the limitation caused by dyspnea in their activities of daily living (ADL) [53].

The Chronic Obstructive Pulmonary Disease (COPD) Assessment Test (CAT) aims to determine the impact of COPD on health status [54]. This questionnaire is simple and easy to apply, consisting of 8 questions about general symptoms and limitation in ADL. The score varies from 0 to 5 points in each item, totaling a maximum of 40 points, and this score has been included in the new classification of patients with COPD, to direct medication treatment and define mortality risk. Lower scores correspond to a low impact of the disease on health status and scores above 10 are representative of patients with higher risk, with poorer health status due to COPD [1]. The Portuguese version will be used, validated for the Brazilian population [55].

#### Sample size

The sample size was calculated using two-way analysis of variance (ANOVA) for repeated measures by analyzing walking distance and the standard deviation in meters on the 6MWT from previously published data [15]. A two-tailed alpha error of 0.01 was implemented with a power of 95%, considering clinical improvement for COPD patients after PR and a difference of 30 m between groups on the walking test. For the calculations, the following means and standard deviations of the time observed in the 6MWT in the rehabilitation and control groups were used: pre-PR 312.43 ± 51.26 and post-PR 342.85 ± 48.82 m (rehabilitation group); pre-PR 305.13 ± 54.60 and post-PR 298.20 ± 52.81 m (control group), and a sample size of 32 subjects was found (effect size = 1.04). As the study of Elci et al. [15] was composed of two groups (rehabilitation and controls) and ours will be composed of three groups, we will assume that the 32 subjects will be part of the rehabilitation program (the PRIMTLL and PRIMTIVH groups), and 16 more subjects will be added as a study control group for a total sample of 48 subjects. In addition, 12 more subjects will be added into the study, because we will also assume a 20% loss to follow-up as well as 5% missing data, for a total sample size of 60 subjects.

#### Monitoring

The research team is responsible for monitoring the study. At each supervised training session, participants will be asked if they have had any changes during the course of their activities, in addition to being monitored with parameters, as described previously, that may be implied in interruption of the activity or withdrawal of the subject from the PR program.

#### Statistics analysis

The Kolmogorov-Smirnov test will be performed to analyze sample normality, and two-way ANOVA followed by a Bonferroni post hoc test will be used to compare the differences among the groups. Data will be analyzed using the GraphPad Prism software version 5.0 (GraphPad Software Inc., San Diego, CA, USA). The significance level was set at 95% (*p* ≤ 0.05).

#### Dissemination

The results found in this study will be presented in specific scientific conferences and will be submitted for publication in peer-reviewed journals.

## Discussion

Literature data reporting the effects of respiratory muscle training regarding exercise tolerance are rare nowadays. We believe that the different protocols used in our study of high load and low load training will be able to guide respiratory therapists to the most effective respiratory muscle training type. Moreover, the study will elucidate the effects of IMT on exercise tolerance, identifying if there is any repercussion on both respiratory muscle static pressure and respiratory muscle strength when the patient is subjected to different efforts.

Finally, the best IMT modality may be included in conventional PR programs, thus optimizing the therapeutic management of COPD patients. We also hope that these findings endorse adjustments to the modalities used for respiratory muscle strengthening, as we believe that a well-implemented therapeutic program may reduce the morbidity/mortality rates for these individuals as well as improve their quality of life.

The protocol performance may show some limitations as individuals’ adherence and subjects’ access to the hospital will vary. Such limitations will be controlled by free specialized medical service and by the provision of a free bus pass allowing subjects to attend the interventions at the PR service. As clinical limitations we may highlight COPD exacerbation and/or other general clinical issues that may lead to a subject’s absence of 3 or more consecutive days or subjects being withdrawn from the study.

### Trial status

The trial is currently in recruitment phase at the Ambulatory Pulmonology department of University Hospital Onofre Lopes (HUOL)/Empresa Brasileira de Serviços Hospitalares (EBSERH). Recruitment will begin in July 2019, and the protocol is expected to finish in June 2020.

## Additional file

Additional file 1: SPIRIT 2013 checklist: recommended items to address in a clinical trial protocol and related documents*. (DOC 121 kb)

## Abbreviations

1RM: 1 maximal repetition
6MWT: Six-Minute Walk Test
ATS/ERS: American Thoracic Society/European Respiratory Society
CAT: COPD Assessment Test
CONSORT: Consolidated Standards of Reporting Trials
COPD: Chronic obstructive pulmonary disease
EBSERH: Empresa Brasileira de Serviços Hospitalares
FEV_1_: Forced expiratory volume in the first second
FVC: Forced vital capacity
GOLD: Global Initiative for Chronic Obstructive Lung Disease
HUOL: University Hospital Onofre Lopes
IMT: Inspiratory muscle training
PRIMTIVH: PR associated with inspiratory muscle training through isocapnic voluntary hyperpnea
PRIMTLL: PR associated with inspiratory muscle training with linear load
ISWT: Incremental Shuttle Walking Test
MEP: Maximal expiratory capacity
MIP: Maximal inspiratory pressure
mmHg: Millimeters of mercury
mMRC: Modified Medical Research Council
OEP: Optoelectronic plethysmography
PR: Pulmonary rehabilitation
PRWIMT: PR associated with inspiratory muscle training without any linear load
RR: Respiratory rate
RV: Residual volume
SAP: Systemic arterial pressure
SNIP: Sniff nasal inspiratory pressure
SPImax: Sustained maximum inspiratory pressure
SPIRIT: Standard Protocol Items: Recommendations for Interventional Trials
SpO_2_: Peripheral oxygen saturation
TLC: Total lung capacity
WHO: World Health Organization
Wmax: Maximum work
